# Defensive Silence, Defensive Voice, Knowledge Hiding, and Counterproductive Work Behavior Through the Lens of Stimulus-Organism-Response

**DOI:** 10.3389/fpsyg.2022.822008

**Published:** 2022-03-18

**Authors:** Fang-Shu Qi, T. Ramayah

**Affiliations:** ^1^School of Management, Universiti Sains Malaysia, Penang, Malaysia; ^2^Information Technology and Management, Daffodil International University, Dhaka, Bangladesh; ^3^Faculty of Economics and Business, Universiti Malaysia Sarawak, Kota Samarahan, Malaysia; ^4^Pusat Kajian Penciptaan Nilai dan Kesejahteraan Insan (INSAN), Fakulti Ekonomi dan Pengurusan (FEP), Universiti Kebangsaan Malaysia (UKM), Bangi, Malaysia; ^5^Fakulti Pengurusan dan Perniagaan, Universiti Teknologi MARA Puncak Alam, Selangor, Malaysia

**Keywords:** defensive silence, defensive voice, knowledge hiding, counterproductive work behavior, stimulus-organism-response (SOR)

## Abstract

Rising negative emotions are like “time bombs” that impede productivity in the workplace. The present investigation provides an insight into the effects of defensive silence and defensive voice on counterproductive work behavior through knowledge hiding in the context of knowledge workers in Chinese academic institutions. Partial least square structural equation modeling (PLS-SEM) was applied to the current samples. The study obtained conjecture the proposed mediating role of knowledge hiding between the negative working attitude and counterproductive work behavior, which is against the organizational norms and performance. The result indicates that the positive relationships exist from defensive silence and defensive voice to counterproductive work behavior, mediated by knowledge hiding. This study links knowledge hiding literature and stimulus-organism-response (SOR) to better explore the academic behavior in a knowledge setting.

## Introduction

The digitalization of diversified information channels portrays volatile knowledge management due to suspicious processing capability and unanalytical elaboration (Fan et al., 2021; Trittin-Ulbrich et al., 2021). However, both individuals and organizations are primarily dependent on the competitive advantage of knowledge management to cope with the dynamic and uncertain environment. The current workplace characterized by increasing competition and decreasing knowledge sharing seeks to dissect the potential risk into the formation of knowledge hiding (Anand et al., 2021). Researchers conceptualize counterproductive knowledge behavior as individuals who intend to conceal knowledge for group members in need (Connelly et al., 2012; Connelly and Zweig, 2015). Hence, investigating the antecedents and consequences of knowledge hiding has gained increased prominence in the priorities of knowledge institutions.

In essence, the radicalized knowledge hiding mirrors unfavorable strategic position as it leads to less team creativity (Fong et al., 2018), contentious work relationships at organizational level through interpersonal conflict (Losada-Otálora et al., 2020), and deviance (Singh, 2019) by considering the employees’ job attitude on empowerment (Offergelt et al., 2019). Therefore, negative emotions related to job attitude can be commonly deemed to be the source of knowledge hiding (Yao et al., 2020b). Even though previous studies have discussed the reasons for knowledge hiding, such as gossip (Yao et al., 2020a), work incivility (Irum et al., 2020), and workplace ostracism (Zhao et al., 2016), limited studies have been conducted to explore the links between both defensive silence and defensive voice to knowledge hiding. This study aimed to fill this literature gap in the context of academic knowledge workers in China.

Academic knowledge workers in academic institutions are often involved in high intensive knowledge activities (Ghani et al., 2020). The main factors that formed knowledge hiding initially become urgent problems to be reckoned with, which would foster greater knowledge interactivity to enhance both sustainable academic and research performance (Chen et al., 2021). Despite this, there was rare research which reveals knowledge hiding toward counterproductive work behavior being fully implemented by knowledge workers.

The present study deepens the predictors of knowledge hiding and indicates its related outcome of counterproductive work behavior through the lens of stimulus-organism-response (SOR). Meanwhile, this study contributes to knowledge hiding literature in various ways. First, specifying the differences of silence and voice under defensive behaviors enables the application of SOR to better explore the antecedents of knowledge hiding. Second, examining the negative consequence of counterproductive work behavior with attempting empirical validation is pressed for further deterrent measures. Third, academic knowledge workers should gregariously self-assured the benefits from ecology-based knowledge setting in the quest for coexistence.

## Literature Review

### Theoretical Background and Hypotheses Development

The theoretical foundation of this study is drawn on the SOR (Mehrabian and Russell, 1974). Rooted in environmental psychology, SOR assumed the outer environment as the stimuli (S) that lead to internal organism (O), which shape the people’s behavior response (R). This model based on SOR was extensively developed to deepen the understanding of knowledge hiding results from defensive silence and defensive voice in emphasizing the negative consequence of counterproductive work behavior.

Stimulus emphasizes the outer environment that is outlined by organizational competence climate to influence the defensive traits of employees. Defensive silence and defensive voice are on describing employee withhold expression because of the fear of negative social opinions. Hence, defensive silence and defensive voice as the predictors of knowledge hiding obtained theoretical support from the role of stimulus.

Organism is associated with knowledge hiding consisting of evasive hiding, playing dumb, and rationalized hiding. Although organism was considered as the important connection part in an SOR model, much remains unknown about employees’ activities that link the relationship between stimulus and response in the knowledge management of academic institutions (Zhai et al., 2020). In this study, knowledge hiding driven by defensive traits on fear to express examined the role of organism in concealing knowledgeable information to workplace members.

Response as the main purpose to develop SOR, describing stimulus and organism may simultaneously lead to significant changes in response. The response converts to behavior, intention, decision, and choice. A number of researchers have examined responses, such as behavior (Kim and Moon, 2009; Siu et al., 2012) and affect (Daunt and Harris, 2012). This study considered counterproductive work behavior as response to investigate how defensive employees conceal knowledge because of fear to express opinions.

**FIGURE 1:**
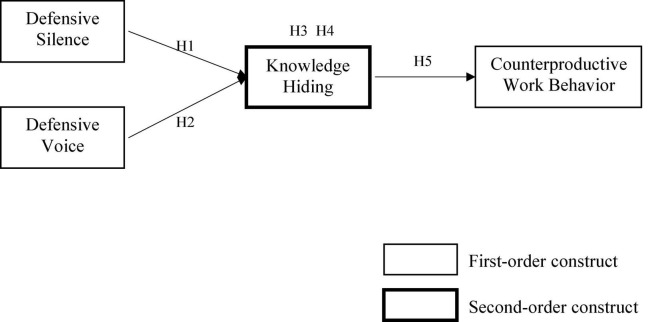
Research model.

In this study, academic knowledge workers with defensive traits (defensive silence and defensive voice) would easily trigger knowledge hiding behavior due to the fear of invading others’ cognitive superiority and disclosure deficiency, which leads to counterproductive work behavior through the disconnect with organizational identity (see Figure 1).

### Defensive Silence and Knowledge Hiding

Defensive silence has been characterized as employees purposely withholding ideas, information, and suggestion at work-related expression (Dyne et al., 2003). Various reasons behind defensive silence have been further explored in knowledge hiding literature. Pinder and Harlos (2001) highlighted employee silence associated with injustice, explaining the reason why employees withhold information. Jahanzeb et al. (2020) elucidated organizational injustice increase knowledge hiding because employees feel disconnected from the organization’s identity. Moreover, many studies revealed psychological safety as the main lens to knowledge behavior, enforcing work focus and reducing worries (Jiang et al., 2019), and boosting harmonious interpersonal trustful climate. On the contrary, scant psychological safety trigger knowledge hiding when employees suffer from intragroup relationship conflict (Peng et al., 2020) and competition (Semerci, 2019). Employees do not express themselves due to psychological insecure that fear negative repercussions. Since limited knowledge management research investigated the defensive silence as the antecedents of knowledge hiding, we propose that:

H1. Defensive silence has a positive relationship with knowledge hiding.

### Defensive Voice and Knowledge Hiding

Defensive voice entails the perceptions of employees intentionally expressing the agreement rather than radicalizing against group (Dyne et al., 2003). Defensive voice focuses on the positive aspect of work-related information rather than accounting for the flaw in problems, diverting attention from workplace criticism. Alternatively, employees typically tend to protect themselves from negative social opinions by refraining from showing their knowledge ability and quality. On this basis, knowledge hiding behavior refers to the concealing or partially sharing knowledge to recipients is motivated by the traits of defensive voice. Meanwhile, the strong effect of leadership on voice behavior decides how employees speak out their opinions with psychological safety (Detert and Burris, 2007). Dark leadership, such as exploitative leadership (Guo et al., 2021) and abusive supervision (Khalid et al., 2018; Pradhan et al., 2019) indulge in interpersonal injustice that employees are involved in aggressive knowledge hiding by defensive voice. Therefore, exploring the defensive voice as a predictor of knowledge hiding remains a significant domain. We hypothesize that:

H2. Defensive voice has a positive relationship with knowledge hiding.

### Mediation of Knowledge Hiding

This study proposes that defensive silence and defensive voice result in counterproductive work behavior by conducting knowledge hiding behavior. In other words, knowledge hiding represents the mediating role of the relationships between defensive traits and negative organizational dynamics. Rooted in SOR, knowledge hiding was stimuli by defensive silence when employees conceal the desire of expression, and often seen as self-protection from negative social opinions. Analogously, defensive voice is conceived as unwilling to attract attention within the group rather than invading others’ cognitive superiority (Unler and Caliskan, 2019). Based on the defensive bondages, employees gained decreased intrinsic drive to disseminate knowledge. Both defensive silence and defensive voice breach employees’ psychology safety (Detert and Burris, 2007; Jiang et al., 2019) that undermines the trust between knowledge disseminators and knowledge recipients. Thereafter, knowledge hiding blocks the chain of knowledge dissemination (Losada-Otálora et al., 2020), leading to a colossal waste of human capital toward the unfavorable work attitude of counterproductive work behavior. Thus, we propose that:

H3. Knowledge hiding mediates the relationship between defensive silence and counterproductive work behavior.

H4. Knowledge hiding mediates the relationship between defensive voice and counterproductive work behavior.

### Knowledge Hiding and Counterproductive Work Behavior

Knowledge economy strategically represents the label of productive forces, as reflected in the pioneers through various industries (Di Vaio et al., 2021). Furthermore, academic knowledge workers have been undertaken voluntary initiatives to the academic civilization and social cognition by knowledge behavior (Ramayah et al., 2014). However, many academic knowledge workers engage in knowledge hiding due to scarce psychological safety (Men et al., 2020), which leads to undesired productive consequences. Literature suggests that knowledge hiding results in counterproductive work behavior wherein employees feel distrust and then inconsistent with a reciprocal role in organization (Arain et al., 2020), adversely affecting their well-being (Khoreva and Wechtler, 2020). Employees who conduct knowledge hiding behaviors may lower task performance (Xiong et al., 2019) by workplace deviance (Singh, 2019). Even though the negative consequences of knowledge hiding have been explored by foregoing discussion, rare empirical study highlights the counterproductive work behavior in academic setting. Therefore, this study accordingly predicts that:

H5. Knowledge hiding has a positive relationship with counterproductive work behavior.

## Measurement

### Sample and Data Collection

The measurement content applied in this study was validated through pre-testing to reduce the response bias, all of the measures were finalized by the means of back-translation procedures (Brislin, 1980). The current sample consists of 460 knowledge workers in Chinese academic institutions. About 57.4% of them are men, 42.6% are women. The largest age group is 36∼45 years old (37.2%). In the terms of educational background, 58.3% of them graduated as masters and 39.6% of them are Ph.D. There are 37% of academic knowledge workers worked less than 5 years. The sciences study field occupies 55.7% compared with the arts study field 44.3%.

### Measures

**Defensive silence and defensive voice** measurements were adopted from Dyne et al. (2003), each with five items ranging from (1) “strongly disagree” to (5) “strongly agree” have been applied in this study.

**Knowledge hiding** was according to Connelly et al. (2012) with five dimensions: evasive hiding, playing dumb, rationalized hiding, lack of sharing, and knowledge hoarding. This study adopted three dimensions of evasive hiding, playing dumb, rationalized hiding to investigate the knowledge hiding behavior within academic relationships. A total of 12 items have been applied through the seven-point Likert scale which is ranging from “strongly disagree” to “strongly agree.”

**Counterproductive work behavior (CWB)** was measured as CWB toward organization from Dalal et al. (2009). The total number of six items was used for the analysis of knowledge workers’ CWB.

## Data Analysis

The structural equation modeling (SEM) analysis comprises measurement model and structural model by applying Smart partial least square (PLS) (Ringle et al., 2015). Common method bias (CMB) associated with the cross-sectional collection panel was based on the different scales of single source data. First, an unmeasured marker variable was used to access the changes of *R*^2^ value after an extra construct arrowed into the research model (Malhotra et al., 2006). The marker variable involved in the difference of *R*^2^ value on the endogenous construct of CWB is 1.1%, which is much less than 10%. Second, according to Kock and Lynn (2012), we tested variance inflation factor (VIF) to eliminate the full collinearity issue. The VIF values of underlying constructs are lower than the threshold of 3.3 (Diamantopoulos and Siguaw, 2006). Overall, the above steps may prove that CMB is not a concern in this study.

### Measurement Model

**FIGURE 2:**
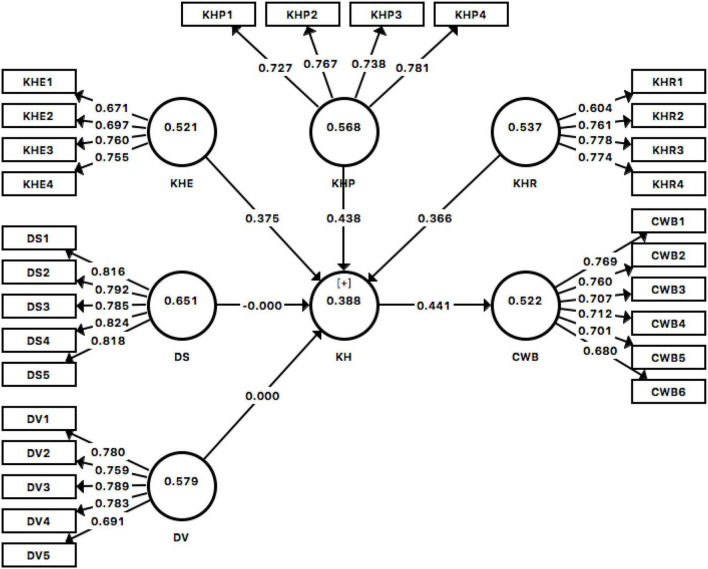
Partial least square (PLS) algorithm result.

The measurement model was assessed through convergent validity, discriminant validity, and reliability. As indicated in Table 1 and Figure 2, all the measurements’ loadings surpassed 0.40 (Hair et al., 2013) and exceeded the cut-off value of average variance extracted (AVE) (Fornell and Larcker, 1981), implying sufficient convergent validity. In addition, the composite reliability of each construct was above the suggested threshold of 0.708 for achieving expected construct reliability (Hair et al., 2013). Another assessment of discriminant validity has been proven through the heterotrait-monotrait ratio of correlations (HTMT) (as shown in Table 2). HTMT was used to estimate the factor correlation and upper limit (Henseler et al., 2015).

**TABLE 1:** The measurement model.

Construct	Items	Loadings	CR	AVE
Defensive silence	DS1	0.816	0.903	0.651
	DS2	0.792		
	DS3	0.785		
	DS4	0.824		
	DS5	0.818		
Defensive voice	DV1	0.780	0.873	0.579
	DV2	0.759		
	DV3	0.789		
	DV4	0.783		
	DV5	0.691		
Evasive hiding	KHE1	0.671	0.813	0.521
	KHE2	0.697		
	KHE3	0.760		
	KHE4	0.755		
Playing dumb	KHP1	0.727	0.840	0.568
	KHP2	0.767		
	KHP3	0.738		
	KHP4	0.781		
Rationalized hiding	KHR1	0.604	0.821	0.537
	KHR2	0.761		
	KHR3	0.778		
	KHR4	0.774		
Counterproductive work behavior	CWB1	0.769	0.867	0.522
	CWB2	0.760		
	CWB3	0.707		
	CWB4	0.712		
	CWB5	0.701		
	CWB6	0.680		

**TABLE 2:** Discriminant validity-heterotrait-monotrait ratio of correlations (HTMT).

	Counterproductive Work behavior	Defensive silence	Defensive voice
**Counterproductive work behavior**			
Defensive silence	0.212		
Defensive voice	0.203	0.515	

This study modeled knowledge hiding as a formative second-order construct that consists of three dimensions of evasive hiding, playing dumb, and rationalized hiding respectively. First, the collinearity test of VIF demotes that the related dimensions are independently forming knowledge hiding behavior. Furthermore, we tested the lower and higher weights of each first-order dimension that aims to reveal the different propensity of knowledge hiding categories. The bootstrapping results illustrated that all three first-order constructs were significantly related to knowledge hiding. Thus, this model included with knowledge hiding is a reflective-formative type II model (Becker et al., 2012) (as shown in Table 3).

**TABLE 3:** Weights of the first-order construct on the designated second-order constructs.

Second-order construct	First-order construct	Measures	Weights	*t*-value	VIF
Knowledge hiding	Evasive hiding	Formative	0.350	3.340	1.839
	Playing dumb	Formative	0.487	4.448	2.152
	Rationalized hiding	Formative	0.336	3.567	1.532

### Structural Model

The bootstrapping method with 5,000 samples was applied in the hypotheses testing. We found that the positive significant relationship between defensive silence and knowledge hiding (ß = 0.267, *t* = 4.880, *p* < 0.001); the positive relationship between defensive voice and knowledge hiding (ß = 0.202, *t* = 3.785, *p* < 0.001); the positive relationship between knowledge hiding and CWB (ß = 0.440, *t* = 10.600, *p* < 0.001). Apart from direct relationships, an indirect relationship of mediation analysis has been supported. The significant mediating role of knowledge hiding exists between defensive silence and CWB (ß = 0.118, *t* = 4.160, *p* < 0.001); the significant mediator of knowledge hiding links defensive voice and CWB (ß = 0.089, *t* = 3.569, *p* < 0.001). Hence, H1, H2, H3, H4, and H5 are all supported and consistent with our prediction (as shown in Table 4).

**TABLE 4:** Hypotheses.

Hypotheses	Relationship	Std. Beta	Std. Dev.	*t*-value	*p*-value	BCI LL	BCI UL	f^2^	VIF	Decision
H1	DS→KH	0.267	0.055	4.880	*p* < 0.001	0.155	0.370	1.362	0.063	Supported
H2	DV→KH	0.202	0.053	3.785	*p* < 0.001	0.100	0.309	1.362	0.036	Supported
H3	DS→KH→CWB	0.118	0.028	4.160	*p* < 0.001	0.058	0.170	–	–	Supported
H4	DV→KH→CWB	0.089	0.025	3.569	*p* < 0.001	0.040	0.138	–	–	Supported
H5	KH→CWB	0.440	0.041	10.600	*p* < 0.001	0.355	0.518	1.000	0.240	Supported

Furthermore, the predictive capacity of structural equation model indicates that knowledge hiding with the *Q*^2^ value of 0.115, CWB with *Q*^2^ value of 0.184. Thus, this model has sufficient predictive relevance (Fornell and Cha, 1994).

## Discussion

In the current study, we address an underexplored boundary condition in the knowledge hiding literature by differentiating defensive silence and defensive voice in understanding focal reasons. The results are congruent with previous literature, which found a positive relationship between defensive silence and knowledge hiding (H1). While employees’ defensive silence influenced by weakened psychological safety evokes employees in knowledge hiding (Semerci, 2019). Besides, workplace ostracism dampens the employees’ beliefs and confidence so that they fortify the potential threat by defensive silence (Chenji and Sode, 2019). Knowledge hiding derives from workplace ostracism highlights the significant role of moral disengagement (Zhao et al., 2016), specifically explaining the link between defensive silence and knowledge hiding. Another finding appears to be that defensive voice for personal purpose at work shows a positive relationship with knowledge hiding (H2). Since defensive voice avoids the idea related to innovation and improvement, erecting a barrier to knowledge management.

Mediation analysis in both paths provides support for the arguments above. This study drew on SOR to justify when knowledge workers perceived defensive traits (e.g., defensive silence and defensive voice), they may have direct response to CWB through knowledge hiding (H3 and H4). Knowledge hiding explained the mechanism from employees’ fear to express toward floppy working attitude. The consequence of knowledge hiding on CWB draws attention for academic knowledge workers (H5). When employees feel misfit with organizational identity, they are more likely to engage in knowledge hiding due to distrust (Zhao et al., 2019). Failing in connecting with the organizational identity, CWB is governed by deficient goals and beliefs (Götz et al., 2020). Hence, a positive relationship exists between knowledge hiding and CWB.

Given previous literature by Connelly et al. (2012), this study empirically validated knowledge hiding as a formative second-order construct that compromises essential ingredients as evasive hiding, playing dumb, and rationalized hiding. The finding suggests that playing dumb is the most significant contributor to knowledge hiding with the highest weightage, followed by evasive hiding and rationalized hiding. The multidimensional construct enables to differentiate various hiding behaviors based on holistic understanding.

### Theoretical Implications

Theoretically, this study validates the SOR model to enrich the existing literature of knowledge hiding, justifying the significant predictors of defensive silence and defensive voice as stimulus (S), knowledge hiding as organism (O), and CWB as response (R) among academic knowledge workers. Findings obtained from this study indicated that knowledge workers with defensive behaviors caused by competitive stress or lack of psychological safety are fear to propose their ideas, information, and suggestions, which are used to avoid potential disputes from external environment. Consequently, academic knowledge workers tend to act CWB through knowledge hiding that impedes knowledge-oriented performance, such as reducing effective knowledge dissemination, leading to inefficient knowledge reciprocation. Hence, SOR in conjunction with the mediating role of knowledge hiding has been supported from the role of response.

### Managerial Implications

The findings of this study have three implications for practicing managers. First, both defensive silence and defensive voice lead to knowledge hiding, which have the detrimental effects on knowledge management. To better encourage knowledge workers from defensive traits, organizations or academic institutions may improve the team psychological safety climate (Qian et al., 2020) and team mastery climate (Černe et al., 2017) through co-creation positive working attitude for fostering knowledge sharing. Second, knowledge hiding exerts the mediating role on CWB. Organizations may process reward systems (Bock et al., 2005), place support on prosocial motivation (Škerlavaj et al., 2018), and ingrain knowledge sharing among academic knowledge workers. Finally, this study shows the consequence of knowledge hiding on CWB, the crucial role of knowledge-oriented leadership might be highlighted to enhance work enthusiasm and professional ethics (Donate and de Pablo, 2015).

### Limitation and Further Suggestion

Considering the several current limitations may leave avenues for potential future attempts. First, the sample limits the generalizability to solely focus on the context of knowledge workers in Chinese academic institutions. Future studies may conduct cross-cultural research to compare the cultural-oriented factors that affect knowledge hiding behavior. Second, although the limitation regarding cross-sectional design is avoided by several tests, such studies could yield longitudinal design to expand causality. Third, specifics related to other boundary conditions and derived by personal differences could be further explored, investigating the potential phenomenon. In addition, further study may investigate knowledge management leadership within a participative climate to enhance the effective knowledge implementation (Pellegrini et al., 2020).

## Data Availability Statement

The raw data supporting the conclusions of this article will be made available by the authors, without undue reservation.

## Author Contributions

F-SQ performed the study conception and design, data collection and analysis, and manuscript. TR commented on the research method and statistical analysis. Both authors contributed to the article and approved the submitted version.

## Conflict of Interest

The authors declare that the research was conducted in the absence of any commercial or financial relationships that could be construed as a potential conflict of interest.

## Publisher’s Note

All claims expressed in this article are solely those of the authors and do not necessarily represent those of their affiliated organizations, or those of the publisher, the editors and the reviewers. Any product that may be evaluated in this article, or claim that may be made by its manufacturer, is not guaranteed or endorsed by the publisher.

## References

[B1] Anand A. Offergelt F. Anand P. (2021). Knowledge hiding–a systematic review and research agenda. *J. Knowl. Manag*. [preprint] 10.1108/JKM-04-2021-0336

[B2] Arain G. A. Bhatti Z. A. Ashraf N. Fang Y. H. (2020). Top-down knowledge hiding in organizations: An empirical study of the consequences of supervisor knowledge hiding among local and foreign workers in the Middle East. *J. Bus. Ethics.* 164 611–625. 10.1007/s10551-018-4056-2

[B3] Becker J. M. Klein K. Wetzels M. (2012). Hierarchical latent variable models in PLS-SEM: Guidelines for using reflective-formative type models. *Long. Range. Plann.* 45 359–394. 10.1016/j.lrp.2012.10.001

[B4] Bock G. W. Zmud R. W. Kim Y. G. Lee J. N. (2005). Behavioral intention formation in knowledge sharing: Examining the roles of extrinsic motivators, social-psychological forces, and organizational climate. *MIS. Quart.* 29 87–111. 10.2307/25148669

[B5] Brislin R. W. (1980). *Translation and content analysis of oral and written material* in Triandis H. C. Berry J. W. Handbook of cross-cultural psychology (Boston, MA.: Allyn & Bacon).

[B6] Černe M. Hernaus T. Dysvik A. Škerlavaj M. (2017). The role of multilevel synergistic interplay among team mastery climate, knowledge hiding, and job characteristics in stimulating innovative work behavior. *Hum. Resour. Manag. J.* 27 281–299. 10.1111/1748-8583.12132

[B7] Chen H. Liu F. Wen Y. Ling L. Gu X. (2021). Compilation and application of the scale of sustainable knowledge sharing willingness in virtual academic community during the times of the coronavirus pandemic (covid-19). *Front. Psychol.* 12:627833. 10.3389/fpsyg.2021.627833 34335355 PMC8322972

[B8] Chenji K. Sode R. (2019). Workplace ostracism and employee creativity: Role of defensive silence and psychological empowerment. *Ind. Commer. Train.* 51 360–370. 10.1108/ICT-05-2019-0049

[B9] Connelly C. E. Zweig D. (2015). How perpetrators and targets construe knowledge hiding in organizations. *Eur. J. Work. Organ. Psy.* 24 479–489. 10.1080/1359432X.2014.931325

[B10] Connelly C. E. Zweig D. Webster J. Trougakos J. P. (2012). Knowledge hiding in organizations. *J. Organ. Behav.* 33 64–88. 10.1002/job.737

[B11] Dalal R. S. Lam H. Weiss H. M. Welch E. R. Hulin C. L. (2009). A within-person approach to work behavior and performance: Concurrent and lagged citizenship-counterproductivity associations, and dynamic relationships with affect and overall job performance. *Acad. Manage. J.* 52 1051–1066.

[B12] Daunt K. L. Harris L. C. (2012). Exploring the forms of dysfunctional customer behaviour: A study of differences in servicescape and customer disaffection with service. *J. Market. Manag.* 28 129–153. 10.5465/amj.2009.44636148

[B13] Detert J. R. Burris E. R. (2007). Leadership behavior and employee voice: Is the door really open? *Acad. Manage. J.* 50 869–884. 10.5465/amj.2007.26279183

[B14] Di Vaio A. Hasan S. Palladino R. Profita F. Mejri I. (2021). Understanding knowledge hiding in business organizations: A bibliometric analysis of research trends, 1988–2020. *J. Bus. Res.* 134 560–573. 10.1016/j.jbusres.2021.05.040

[B15] Diamantopoulos A. Siguaw J. A. (2006). Formative versus reflective indicators in organizational measure development: A comparison and empirical illustration. *Brit. J. Manage.* 17 263–282. 10.1111/j.1467-8551.2006.00500.x

[B16] Donate M. J. de Pablo J. D. S. (2015). The role of knowledge-oriented leadership in knowledge management practices and innovation. *J. Bus. Res.* 68 360–370. 10.1016/j.jbusres.2014.06.022

[B17] Dyne L. V. Ang S. Botero I. C. (2003). Conceptualizing employee silence and employee voice as multidimensional constructs. *J. Manage. Stud.* 40 1359–1392. 10.1111/1467-6486.00384

[B18] Fan M. Huang Y. Qalati S. A. Shah S. Ostic D. Pu Z. (2021). Effects of information overload, communication overload, and inequality on digital distrust: A cyber-violence behavior mechanism. *Front. Psychol.* 12:643981. 10.3389/fpsyg.2021.643981 33959073 PMC8093436

[B19] Fong P. S. W. Men C. Luo J. Jia R. (2018). Knowledge hiding and team creativity: The contingent role of task interdependence. *Manage. Decis.* 56 329–343. 10.1108/MD-11-2016-0778

[B20] Fornell C. Cha J. (1994). “Partial least squares,” in *Advanced Methods of Marketing Research*, ed. Bagozzi R. P. (Cambridge: Blackwell), 52–78.

[B21] Fornell C. Larcker D. F. (1981). Evaluating structural equation models with unobservable variables and measurement error. *J. Marketing. Res.* 18 39–50. 10.1177/002224378101800104

[B22] Ghani U. Zhai X. Spector J. M. Chen N. S. Lin L. Ding D. (2020). Knowledge hiding in higher education: Role of interactional justice and professional commitment. *High. Educ.* 79 325–344. 10.1007/s10734-019-00412-5

[B23] Götz M. Donzallaz M. Jonas K. (2020). Leader–member exchange fosters beneficial and prevents detrimental workplace behavior: Organizational identification as the linking pin. *Front. Psychol.* 11:1788. 10.3389/fpsyg.2020.01788 33013499 PMC7461862

[B24] Guo L. Cheng K. Luo J. (2021). The effect of exploitative leadership on knowledge hiding: A conservation of resources perspective. *Leadership. Org. Dev. J.* 42 83–98. 10.1108/LODJ-03-2020-0085

[B25] Hair J. F. Ringle C. M. Sarstedt M. (2013). Partial least squares structural equation modeling: Rigorous applications, better results and higher acceptance. *Long. Range. Plann.* 46 1–12. 10.1016/j.lrp.2013.01.001

[B26] Henseler J. Ringle C. M. Sarstedt M. (2015). A new criterion for assessing discriminant validity in variance-based structural equation modeling. *J. Acad. Market. Sci.* 43 115–135. 10.1007/s11747-014-0403-8

[B27] Irum A. Ghosh K. Pandey A. (2020). Workplace incivility and knowledge hiding: A research agenda. *BIJ.* 27 958–980. 10.1108/BIJ-05-2019-0213

[B28] Jahanzeb S. De Clercq D. Fatima T. (2020). Organizational injustice and knowledge hiding: The roles of organizational dis-identification and benevolence. *Manage. Decis.* 59 446–462. 10.1108/MD-05-2019-0581

[B29] Jiang Z. Hu X. Wang Z. Jiang X. (2019). Knowledge hiding as a barrier to thriving: The mediating role of psychological safety and moderating role of organizational cynicism. *J. Organ. Behav.* 40 800–818. 10.1002/job.2358

[B30] Khalid M. Bashir S. Khan A. K. Abbas N. (2018). When and how abusive supervision leads to knowledge hiding behaviors: An Islamic work ethics perspective. *Leadership. Org. Dev. J.* 39 794–806. 10.1108/LODJ-05-2017-0140

[B31] Khoreva V. Wechtler H. (2020). Exploring the consequences of knowledge hiding: An agency theory perspective. *J. Manage. Psychol.* 35 71–84. 10.1108/JMP-11-2018-0514

[B32] Kim W. G. Moon Y. J. (2009). Customers’ cognitive, emotional, and actionable response to the servicescape: A test of the moderating effect of the restaurant type. *Int. J. Hosp. Manag.* 28 144–156. 10.1016/j.ijhm.2008.06.010

[B33] Kock N. Lynn G. (2012). Lateral collinearity and misleading results in variance-based SEM: An illustration and recommendations. *J. Assoc. Inf. Syst.* 13 546–580.

[B34] Losada-Otálora M. Peña-García N. Sánchez I. D. (2020). Interpersonal conflict at work and knowledge hiding in service organizations: The mediator role of employee well-being. *Int. J. Qual. Serv. Sci.* 13 63–90. 10.1108/IJQSS-02-2020-0023

[B35] Malhotra N. K. Kim S. S. Patil A. (2006). Common method variance in IS research: A comparison of alternative approaches and a reanalysis of past research. *Manag. Sci.* 52 1865–1883. 10.1287/mnsc.1060.0597 19642375

[B36] Mehrabian A. Russell J. A. (1974). *An approach to environmental psychology.* Cambridge: the MIT Press.

[B37] Men C. Fong P. S. W. Huo W. Zhong J. Jia R. Luo J. (2020). Ethical leadership and knowledge hiding: A moderated mediation model of psychological safety and mastery climate. *J. Bus. Ethics.* 166 461–472. 10.1007/s10551-018-4027-7

[B38] Offergelt F. Spörrle M. Moser K. Shaw J. D. (2019). Leader-signaled knowledge hiding: Effects on employees’ job attitudes and empowerment. *J. Organ. Behav.* 40 819–833. 10.1002/job.2343

[B39] Pellegrini M. M. Ciampi F. Marzi G. Orlando B. (2020). The relationship between knowledge management and leadership: mapping the field and providing future research avenues. *J. Knowl. Manag.* 24 1445–1492. 10.1108/jkm-01-2020-0034

[B40] Peng H. Bell C. Li Y. (2020). How and when intragroup relationship conflict leads to knowledge hiding: The roles of envy and trait competitiveness. *Int. J. Confl. Manage.* 32 383–406. 10.1108/IJCMA-03-2020-0041

[B41] Pinder C. C. Harlos K. P. (2001). “Employee silence: Quiescence and acquiescence as responses to perceived injustice,” in *Research In Personnel And Human Resources Management*, Vol. 20 eds Rowland K. M. Ferris G. R. (New York: Emerald Group Publishing Limited), 331–369.

[B42] Pradhan S. Srivastava A. Mishra D. K. (2019). Abusive supervision and knowledge hiding: The mediating role of psychological contract violation and supervisor directed aggression. *J. Knowl. Manag*. 24 216–234. 10.1108/JKM-05-2019-0248

[B43] Qian J. Zhang W. Qu Y. Wang B. Chen M. (2020). The enactment of knowledge sharing: The roles of psychological availability and team psychological safety climate. *Front. Psychol.* 11:2292. 10.3389/fpsyg.2020.551366 33071870 PMC7538610

[B44] Ramayah T. Yeap J. A. L. Ignatius J. (2014). Assessing knowledge sharing among academics: A validation of the knowledge sharing behavior scale (KSBS). *Evaluation. Rev.* 38 160–187. 10.1177/0193841X14539685 25015259

[B45] Ringle C. M. Wende S. Becker J. M. (2015). *”SmartPLS 3.” Boenningstedt: SmartPLS GmbH.* Available online at: http://www.smartpls.com/ (accessed September, 2021).

[B46] Semerci A. B. (2019). Examination of knowledge hiding with conflict, competition and personal values. *Int. J. Confl. Manage.* 31 111–131. 10.1108/IJCMA-03-2018-0044

[B47] Singh S. K. (2019). Territoriality, task performance, and workplace deviance: Empirical evidence on role of knowledge hiding. *J. Bus. Res.* 97 10–19. 10.1016/j.jbusres.2018.12.034

[B48] Siu N. Y. M. Wan P. Y. K. Dong P. (2012). The impact of the servicescape on the desire to stay in convention and exhibition centers: The case of Macao. *Int. J. Hosp. Manag.* 31 236–246. 10.1016/j.ijhm.2011.06.011

[B49] Škerlavaj M. Connelly C. E. Cerne M. Dysvik A. (2018). Tell me if you can: Time pressure, prosocial motivation, perspective taking, and knowledge hiding. *J. Knowl. Manag*. 22 1489–1509. 10.1108/JKM-05-2017-0179

[B50] Trittin-Ulbrich H. Scherer A. G. Munro I. Whelan G. (2021). Exploring the dark and unexpected sides of digitalization: Toward a critical agenda. *Organ.* 28 8–25. 10.1177/1350508420968184

[B51] Unler E. Caliskan S. (2019). Individual and managerial predictors of the different forms of employee voice. *J. Manag. Dev.* 38 582–603. 10.1108/JMD-02-2019-0049

[B52] Xiong C. Chang V. Scuotto V. Shi Y. Paoloni N. (2019). The social-psychological approach in understanding knowledge hiding within international R&D teams: an inductive analysis. *J. Bus. Res.* 128 799–811. 10.1016/j.jbusres.2019.04.009

[B53] Yao Z. Zhang X. Luo J. Huang H. (2020b). Offense is the best defense: The impact of workplace bullying on knowledge hiding. *J. Knowl. Manag*. 24 675–695. 10.1108/JKM-12-2019-0755

[B54] Yao Z. Luo J. Zhang X. (2020a). Gossip is a fearful thing: The impact of negative workplace gossip on knowledge hiding. *J. Knowl. Manag*. 24 1755–1775. 10.1108/JKM-04-2020-0264

[B55] Zhai X. Wang M. Ghani U. (2020). The SOR (stimulus-organism-response) paradigm in online learning: An empirical study of students’ knowledge hiding perceptions. *Interact. Learn. Envir.* 28 586–601. 10.1080/10494820.2019.1696841

[B56] Zhao H. Liu W. Li J. Yu X. (2019). Leader–member exchange, organizational identification, and knowledge hiding: The moderating role of relative leader–member exchange. *J. Organ. Behav.* 40 834–848. 10.1002/job.2359

[B57] Zhao H. Xia Q. He P. Sheard G. Wan P. (2016). Workplace ostracism and knowledge hiding in service organizations. *Int. J. Hosp. Manag.* 59 84–94. 10.1016/j.ijhm.2016.09.009

